# Less extrusion debris during the retreatment of curved canals using twisted files with higher rotational speeds: an *ex vivo* study

**DOI:** 10.1186/s12903-017-0340-2

**Published:** 2017-01-16

**Authors:** Mengdong Liu, Shijiang Xiong, Fei Tan, Yi Liu

**Affiliations:** 1 School of Dentistry, Shandong University, 44, Wenhua West Road, Jinan, 250012 Shandong Province China; 2 School of Stomatology, Qingdao University, Qingdao, 266021 Shandong Province China

**Keywords:** Apical debris, Root canal therapy, Rotational speed, Tooth apex, Triple-Flex Files, Twisted Files

## Abstract

**Background:**

Debris extrusion from the apical foramen can be problematic in severely curved canals. This study aimed to assess the use of Twisted Files (TF) at different rotational speeds during retreatment, compared with manual technique using Triple-Flex Files (TFF).

**Methods:**

Forty-eight mesiobuccal root canals were randomized to four groups (*n* = 12 per group). In groups A, B, and C, gutta-percha was removed using TF at 500, 1000, and 1500 rpm, respectively, while it was removed using TFF in group D. Apical debris was collected in a pre-weighed centrifuge tube. The weight of dry debris was assessed by comparing the pre- and post-instrumentation weight for each group.

**Results:**

Instrumentation in group D (0.69 ± 0.04 mg) extruded significantly more debris than any of the TF groups (A: 0.54 ± 0.05 mg; B: 0.48 ± 0.04 mg; C: 0.42 ± 0.03 mg; all *P* < 0.001). In addition, increasing the rotational speed of TF decreased the amount of extruded debris (A vs. B: *P* = 0.006; B vs. C: *P* < 0.001; A vs. C: *P* < 0.001).

**Conclusions:**

TF at 1500 rpm produced less apical extrusion debris than other TF operating speeds and TFF.

## Background

The main goal of endodontic retreatment is to remove infected dentin and root-filling material throughout the canal length to re-establish decontamination of the root canal system and to preserve the tooth [1, 2]. Debris generated during retreatment may cause undesired consequences, such as inflammation and postoperative pain [1, 3]. Many techniques have been used to remove gutta-percha in root-filled teeth, such as hand files, heat, solvents, and rotary instruments [4]. Unfortunately there is considerable evidence that all of these techniques apically extrude some of the debris, but the amount is likely to vary according to the technique used [4–6]. Motor-driven rotary devices are associated with less dentinal debris extrusion because of their unique file design that also works as an Archimedic screw [7], but the complexity of root canal anatomy makes retreatment difficult and highlights the need for investigation of these issues. Indeed, when using rotary instruments, the high degree of root canal curvature may lead to iatrogenic damage, and this risk in increased with higher rotational speed. A proper evaluation of the different tools available would then allow appropriate and safe retreatment techniques to be selected with the lowest incidence of extrusion possible.

Twisted Files (TF; SybronEndo, Orange, CA, USA) are rotary nickel titanium endodontic files that were introduced in 2008. They can be twisted and converted back into an austenite structure by heating and cooling [8]. The manufacturer claims that TF are superior to traditional file systems due to their cyclic fatigue resistance, flexibility, and better cutting efficiency [9]. Compared with other instruments, TF can work safely at higher rotational speeds, but few studies have investigated the behavior of TF during retreatment [10]. Previous retreatment studies have been performed on relatively straight root canals [11–13], which may not accurately represent the challenge of retreating curved canals. Furthermore, almost all rotary instruments were studied at similar speeds. Therefore, the influence of rotational speed on effective retreatment has not been assessed.

The purpose of the present study was to quantitatively evaluate the amount of debris extruded apically after endodontic retreatment with TF at different operating speeds, compared with traditional hand techniques using Triple-Flex Files in severely curved root canals.

## Methods

### Specimen selection

A total of 48 mandibular first molars were collected from patients with periodontal disease undergoing tooth extraction. To be included in the study, tooth had to be without a history or disease or treatment. This study was approved by the ethics committee of the Affiliated Hospital of Qingdao University.

Radiographs of each tooth were obtained, digitized, and stored electronically using the Digora Optime digital imaging system (Soredex, Tuusula, Finland). Angles and radius of curvature were measured using the proprietary software Digora for Windows (DfW version 2.7, Soredex, Tuusula, Finland). Root canal curvature was determined according to the Schneider’s method [14]. The radius of each sample was measured according to Schäfer et al. [15].

The mesiobuccal root canals were used when encountering the following criteria: 1) curvature ranging between 25° and 35°; 2) a radius below 10 mm in a buccal direction; and 3) not more than 5° in a mesial to distal direction. The mesiobuccal root canals were examined under an operating microscope (Carl Zeiss, Jena, Germany) to verify the presence of a single apical foramen, with fully formed apices. The roots had no previous root canal treatment. The access cavity was prepared using high-speed diamond burs (Dentsply Maillefer, Ballaigues, Switzerland) with water cooling. After accessing the cavity, the canal patency was established with a size 10 K-file (Dentsply Maillefer, Ballaigues, Switzerland). Only canals with an initial apical size equivalent to a size 10 K-file were selected.

According to the above criteria, 48 molars with severely curved mesiobuccal root canals were collected and thoroughly cleaned by removing the calculus and the soft deposits using curettes. The teeth were stored in a saline solution before use.

### Root canal preparation and obturation

Apical patencies were determined with a size 10 K-file. The K-file was introduced carefully into the canal until it was visible at the apical foramen, and the working length (WL) was established as 1 mm short of this length. The crowns were flattened, so that a final dimension of 13-mm working length was achieved for each root.

The coronal portion was initially flared with Gates Glidden drills (Dentsply Maillefer, Ballaigues, Switzerland) sizes 1–3 and was instrumented using ProTaper Universal Rotary Files (Dentsply Maillefer, Ballaigues, Switzerland) up to size F1, according to the manufacturer’s instructions. Each instrument was used for the preparation of only one tooth. On withdrawal of each instrument, the canals were flushed with 5 mL of distilled water. ProTaper Universal Gutta Percha Point size F1 (Dentsply Maillefer, Ballaigues, Switzerland) was selected as the master gutta-percha cone fitted with tug-back at the WL. The root canals were dried with sterile paper points and obturated with the master cone and sealer using a continuous wave of condensation technique (System B, Elements Obturation Unit, Sybron Endo, Orange, CA, USA). The total length of the root canal filling did not exceed the level of the staging platform from the apex to the coronal aspect, and also assessed by anteroposterior and lateral view X-ray photography, in an attempt to control the amount of root filling in each tooth, so that the volume of the filling material was approximately equal in all root canals.

The teeth were radiographed (Soredex Minray, Soredex, Milwaukee, WI, USA) at different angles to confirm the quality of the filling procedure. The access cavities were temporarily filled (Cavition-GC, Tokyo, Japan). The obturated roots were stored in 100% humidity at 37°C for two weeks to allow complete setting of the sealer.

### Experimental process

**Fig. 1:**
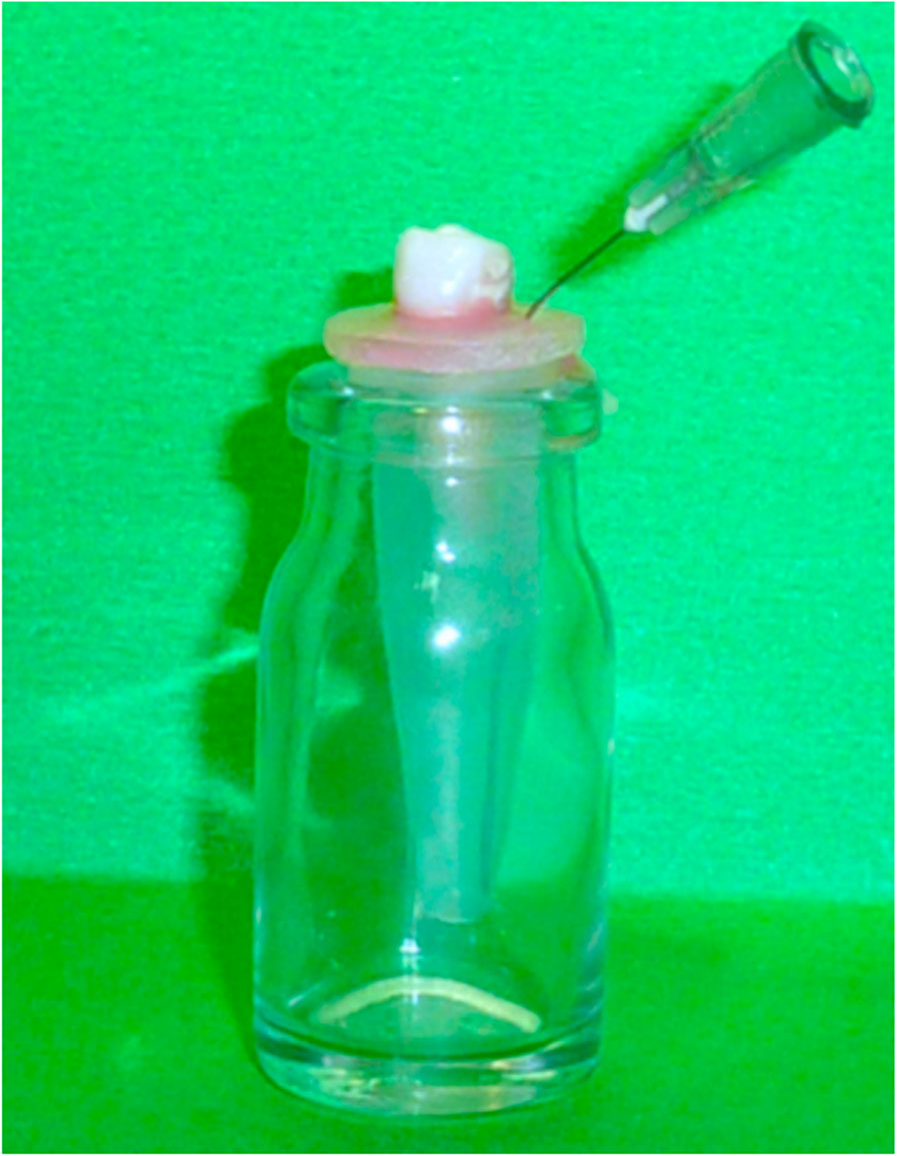
Collection instrument used to evaluate the apically extruded debris

The instrument used to evaluate the extruded debris was a minor adaptation from a previously used design (Fig. 1) [16]. The teeth were held securely under pressure by stoppers, and the root apex was hung within the receptor tube and suspended below the upper rim of the centrifuge tube. A bent 27-gauge needle was forced alongside the stopper to use as a drainage cannula and to balance the air pressure inside and outside the tubes. The tube was held vertically by hand only by the outer vial during retreatment. In no case was the inner tube touched by the operator’s fingers. All vials were covered with adhesive plaster to prevent the operator from viewing debris extrusion during retreatment. The tubes were individually preweighed three times with a 10^−5^ g precision analytic microbalance (Sartorius AG, Göttingen, Germany).

### Retreatment technique

All teeth were coded and randomized to four groups (12 teeth/group). Gutta-percha in the coronal portion was removed using size 1–3 Gates Glidden drills. The rotary instrument was driven by an endodontic motor (NSK, Endo-Mate DT, Tokyo, Japan) with the crown-down technique at the recommended speed and torque according to the manufacturer’s instructions. The final apical file was one size larger than that used in the first preparation. The four groups were then retreated according to their group allocation. No apical counter pressure was applied.

Group A: Specimens were retreated using TF at 500 rpm without torque-control, according to the manufacturer’s recommendations, and using a 4:1 speed reduction and an electric motor at 2000 rpm. The instrumentation sequence was .01/#25, .06/#25, and .08/#25 at the WL. The shaping procedure began with TF size .04/#25. TF size 25/08 was inserted and used as the final apical file.

Group B: TF at 1000 rpm. The method for gutta-percha removal was the same as in group A except the motor was used with 4:1 speed reduction and was powered by an electric motor at 4000 rpm, resulting in instrument rotation speed of 1000 rpm.

Group C: TF at 1500 rpm. The method for gutta-percha removal was the same as in group A except the motor was used with 4:1 speed reduction and was powered by an electric motor at 6000 rpm, resulting in instrument rotation of 1500 rpm.

Group D: manual technique. The canals were reinstrumented sequentially using triangular Triple-Flex Files (SybronEndo, Orange, CA, USA; made of stainless steel designed for flexibility and integrity) in sizes 20 to 25, to a master apical file size of 25. The files were pre-curved according to root canal direction and gutta-percha was removed from the canal using the balance force technique and step-back in 0.625-mm increments (determined using a caliper) to a file size 55 so that the taper was determined to be 8%. Canal patency was maintained with a size 25 K-file during reinstrumentation. Consequently, the final canal diameter and taper was comparable to the samples in the other groups.

### Debris collection

During all retreatment procedures, the flutes of the files were cleaned completely after each use. Canals were flushed with 20 mL of distilled water. All instruments were used for only one specimen and then discarded. Any deformed instruments were also discarded. Retreatment was deemed complete when the WL was reached and no more gutta-percha was visible on the last instrument used in every group, based on the step-back approach [17].

After retreatment, any debris (dentin and filling material) visually adherent to the external surface of the apex was scraped and collected into the tube containing the sample. The root apex was flushed with 0.5 mL of distilled water to wash any remaining debris (dentin and filling material) into its tube. No solvent was used. Each root canal was prepared, filled, and retreated by the same operator to reduce interoperator variability.

After the retreatment procedure, the centrifuge tubes were removed from the vials by handling with clean cotton forceps at all times. They were stored in an incubator at 37°C for 4 weeks to evaporate moisture before finally being weighed three times on a microbalance and the average value was used for analysis. The weight of the extruded debris was determined by subtracting the weight of the preweighed empty tube from the final weight. Evaluation was carried out by a second examiner who was blinded to group assignment.

### Statistical analysis

Statistical analysis was performed using SPSS 19.0 (IBM, Armonk, NY, USA). In the preliminary analysis, the raw data followed a normal distribution (Shapiro-Wilk test); statistical analysis was thus performed using parametric one-way analysis of variance. Post-hoc pairwise comparisons were performed using the Tukey’s multiple comparison test. Statistical significance was established at 0.05.

## Results

### Characteristics of the teeth

Table 1 presents the angle and radius of curvature, respectively, of the 48 teeth of the four groups. Results showed that the four groups were similar (all *P* > 0.05).

**Table 1:** Angle and radius of canal curvature of the 48 teeth

	Angle			Radius		
Group	Mean angle (degree)	SD	Range	Mean radius (mm)	SD	Range
A(*n* = 12)	28.95	3.15	25.1–35.0	6.82	2.17	3.0–9.8
B(*n* = 12)	34.90	29.3	25.1–34.9	6.86	1.83	3.7–9.8
C(*n* = 12)	31.05	2.73	27.6–35.0	7.25	2.15	3.9–10.0
D(*n* = 12)	29.43	2.99	25.0–34.0	7.21	2.21	3.2–10.0

Group A: Twisted Files at 500 rpm; Group B: Twisted Files at 1000 rpm; Group C: Twisted Files at 1500 rpm; Group D: manual technique

*SD* standard deviation

### Amount of debris

The amount of apical debris for each group is shown in Table 2. The results indicated that all retreatment techniques used in this study caused measurable apical extrusion of debris. The manual instrumentation group (group D) extruded significantly more debris (0.69 ± 0.04 mg) than the TF groups (A: 0.54 ± 0.05 mg; B: 0.48 ± 0.04 mg; C: 0.42 ± 0.03 mg; all *P* < 0.001). In addition, increasing the rotational speed of TF decreased the amount of extruded debris (A vs. B: *P* = 0.006; B vs. C: *P* < 0.001; A vs. C: *P* < 0.001).

**Table 2:** Amount of Apically Extruded Debris

Group	Number	Mean Weight (mg)	SD	Range
A	12	0.54	0.05	0.45–0.61
B	12	0.48	0.04	0.42–0.56
C	12	0.42	0.03	0.38–0.49
D	12	0.69	0.04	0.62–0.76

Group A: Twisted Files at 500 rpm; Group B: Twisted Files at 1000 rpm; Group C: Twisted Files at 1500 rpm; Group D: manual technique

*SD* standard deviation

## Discussion

The aim of this study was to evaluate the use of TF for endodontic retreatment in terms of prevention of extrusion debris and in cases of severely curved mesiobuccal root canals. To do so, three different speeds of rotation were compared when using the TF and manual treatment of curved root canals. The results showed that each of the methods used produced extrusion debris, but that TF at 1500 rpm produced the smallest amount.

Reducing extrusion debris is important for effective endodontic retreatment as it reduces the likelihood of inflammation and postoperative pain. These results are consistent with previous retreatment studies that used various nickel titanium rotary instruments compared with conventional instruments, and demonstrated that rotary instruments produced less debris [12]. This is due, at least in part, to the up-and-down motion of the manual files, which is more likely to push debris toward the apical end [12].

Previous studies on retreatments have been performed on relatively straight root canals [11–13], because their low curvatures eliminated complications likely to arise in the instrumentation of severely curved root canals. However, it can be argued that the filling materials in straight root canals are more easily removed so that canal repreparation tends to extrude less debris, and this may not accurately represent the challenge of retreating curved canals. On the contrary, teeth with high degrees of curvature may give rise to different results [18]. Therefore, studying these devices in straight root canals only does not fully reflect the differences among various technologies. It has been shown that in mandibular first molars’ mesiobuccal root canals the mean curvature is 25° and the mean radius is 10.6 mm [15]. Thus, the present study investigated mesiobuccal root canals with severe curvature angles of 25–35° and a short radius of <10 mm in an attempt to simulate these challenging clinical conditions. To the best of the authors’ knowledge, there have been no peer-reviewed studies in which molars with severely curved roots were used to assess the amount of dentin debris extruded during retreatment.

In previous studies, the continuous wave of condensation technique [19] has been rarely used in curved canals. We used this method before retreatment to ensure filling and to avoid filling defects, to make that the results of the investigation are more reliable. The technique has become increasingly popular because it uses heat to produce a homogenous obturation that adapts well to the canal walls and replicates the prepared root-canal space [20, 21].

Unlike previous studies, a major factor in the present study was the use of different rotational speeds. High rotational speed not only increases efficiency but also probably produces more heat [22]. This may be because rotary reinstrumentation generates frictional heat during its contact with gutta-percha and dentin. In addition, rotational speed is associated with heat generation during the formation of martensite [23]. The resulting heat could plasticize the gutta-percha, making it into a single block, thus facilitating its removal and minimizing extruded debris [24]. In addition, plasticized gutta-percha cannot pass through the apical foramen [24], which could have contributed to the differences observed at high rotational speed. Furthermore, the Archimedes screw effect due to higher rotational speed should be taken into account [25]. On the other hand, the exact same methods for coronal introduction of gutta-percha was used among all groups, and eventual differences in pressure should be counterbalanced by the standard method and randomization of the samples. Nevertheless, the plasticization of gutta-percha due to heat during retreatment could be associated with thermal damage to the teeth [26], but heat damage was not examined in the present study. Additional studies are necessary to determine if heat due to rotational speed during retreatment is associated with significant heat damage.

Although the current ground-fluted rotary instruments that are commonly used have excellent performance, there are inevitably many drawbacks due to their design. They have difficulties negotiating a severe curvature, which may increase the risk of iatrogenic damage such as bleeding and perforation of the lateral wall of the root canal. Furthermore, they must be operated in strict accordance with the speed and torque instructions, otherwise there is a risk of unexpected instrument separation, especially in curved root canals [27]. Since most devices have similar low rotational speeds, and the difference is very small, comparisons between device speeds are almost meaningless. Rotational devices that can be used effectively in curved root canals and at higher speeds could improve retreatment.

TF, which are manufactured by twisting instead of grinding the nickel titanium alloy, have been shown to have excellent performance [8]. TF with R-phase technology gives a much higher level of flexibility and can perform side-cutting with great efficiency, while still successfully negotiating a complex curvature. Above all, TF can work at speeds up to 1500 rpm, underlining that using higher rotational speed will lead to less debris. The manufacturer suggests that using a speed of 500 rpm is likely to reduce waste and increase the service life of the instrument, but our experience of using it between 900 and 1500 rpm is also positive and more efficient. We therefore tested TF at three different speeds 500, 1000, and 1500 rpm to assess whether speed would influence the amount of apical debris. The results showed that TF at the highest rotational speed produced the least debris. The results of the experiment were consistent with our inference of the benefits of increased speed and temperature. Nevertheless, other instruments use different rotational speeds and additional studies are necessary to assess these instruments.

This study has some limitations. The clinical relevance of this ex vivo study should be interpreted with caution. In vivo, the apex would be surrounded by periradicular tissues that may serve as a natural barrier preventing debris extrusion. In an early (1977) in vivo study, Salzgeber and Brilliant [28] showed that vital tissues helped control the apical and lateral penetration of an irrigating solution. In addition to the apical control of extruded debris, other factors involved in retreatment require further evaluation. For instance, the heat produced by high speed rotation may be transmitted to the outer root. It has been shown that a 10°C rise may cause irreversible thermal damage to the supporting periodontal structures [29]. Whether the heat produced in this investigation could cause damage to the surrounding tissue needs further evaluation. Finally, apart the power tool nature of TF, there was intrinsic differences between the TF and the manual instrument, such as the cross section (round for TF and triangular for manual files), alloy, and the number of files used. Nevertheless, groups A, B, and C used the same instrument. Additional research will be necessary to evaluate the cleaning efficiency of the TF at higher speeds and to determine the optimal rotational speeds (and torque values) for the removal of gutta-percha.

## Conclusions

These results provide important information on preventing apical debris and should help improve retreatment outcomes. TF at high rotational speed was associated with less debris extrusion than at lower speeds, and in comparison with manual endodontic retreatment in curved root canals. Speed may be a significant factor for the retreatment results and may improve retreatment outcomes. Further research is needed to fully evaluate the effect of high rotational speed on endodontic retreatment.

## Abbreviations

TF: Twisted files
TFF: Triple-flex files
WL: Working length
